# The Impact of the COVID-19 Pandemic on the Composition of Dietary Supplements and Functional Foods Notified in Poland

**DOI:** 10.3390/ijerph182211751

**Published:** 2021-11-09

**Authors:** Kacper Wróbel, Anna Justyna Milewska, Michał Marczak, Remigiusz Kozłowski

**Affiliations:** 1 Department of Management and Logistics in Healthcare, Medical University of Lodz, 90-131 Lodz, Poland; michal.marczak@umed.lodz.pl; 2 Department of Statistics and Medical Informatics, Medical University of Bialystok, 15-089 Bialystok, Poland; anna.milewska@umb.edu.pl; 3 Department of Emergency Medicine and Disaster Medicine, Medical University of Lodz, 92-212 Lodz, Poland; remigiusz.kozlowski@umed.lodz.pl

**Keywords:** dietary supplements, COVID, vitamins, minerals

## Abstract

The COVID-19 pandemic has exerted a strong impact on numerous areas of everyday life. The aim of this study was to check how the pandemic influenced the composition of dietary supplements and other functional food products placed on the market till March 2021, compared to 2019. For this purpose, data concerning the registered products and reports of popularity of online searches of terms connected with vitamins and minerals were used. The results of the study made it possible to determine the group of ingredients especially popular during the pandemic. Their use in products after the announcement of the pandemic was significantly higher than in the preceding period. In conclusion, it can be shown that the pandemic changed the ingredients used in functional foods—mainly as far as vitamins and minerals are concerned. The highest proportional increase in its use in dietary supplements was noted for potassium. Personalized therapy has also become more popular, promoted by one of the manufacturers of dietary supplements active during the pandemic. Moreover, different phases of the pandemic were characterized by the popularity of different ingredients among the consumers—first, these were immunity-boosting ingredients, then those that improved psychological functions, and finally mixtures with universal health effects.

## 1. Introduction

Legally, dietary supplements were defined in Poland for the first time in 2001, in the Act on sanitary conditions of food and nutrition. At that point, this group of food products was described as a concentrated source of vitamins or minerals as well as other nutrients. The ingredients could occur individually or in combination as composite products. Their role was to supplement the consumption of nutrients that are part of the normal diet [1].

Over the next 18 years, the definition of dietary supplements was reformulated and clarified at least twice; however, despite the passing of years, it did not lose its essential character [2,3]. Doubtlessly, the market of dietary supplements in Poland has been in a state of dynamic development, increasing its value each year, while the products offered in this group of foods differ significantly from those offered at the beginning of the century.

Soon after a group of patients with symptoms of pneumonia that eventually turned out to be the symptoms of infection with a new type of virus, i.e., SARS-CoV-2, were diagnosed in China in December 2019, the European markets of dietary supplements reported growths [4]. Just over three months later, on 4 March 2020, the first case of COVID-19 infection caused by the aforementioned virus was diagnosed in Poland [5].

In the analysed period, 1,998,733 cases of COVID-19 infection were diagnosed in Poland, with only 6 cases confirmed in the week preceding the announcement of the pandemic. Newly diagnosed cases accumulated in the 46th week of the year.

**Figure 1:**
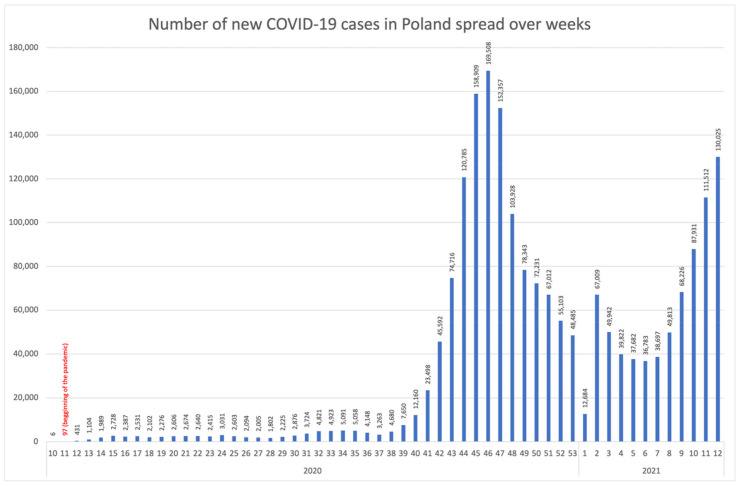
Number of new COVID-19 cases in Poland spread over weeks.

In the analysed period in Poland, two waves of infection may be recognized. The first occurred in the period between 4 March and 31 May 2020 [6], with its peak between approx. 1 to 20 April 2020, when the most severe social and economic restrictions were implemented [7]. The time frame of the first wave does not result directly from the number of infections, yet it shows some correlation with the number of deaths in certain countries —in Poland an increase in the number of deaths among men was observed in the 16th week of 2020 [8]. Despite the relatively low number of infections, an increase in health anxiety was observed in the society, which manifested itself, e.g., in the growing popularity of health-related Internet searches [9]. This study adopts the period between the 14th and the 17th week of the year as the wave peak. The second wave is closely linked with the growing number of COVID-19 infections and covers the period from October 1 up until the end of the year [6], with the peak occurring from 3 to 29 November 2020, which corresponds to the period between the 45th and 49th week of the year [10] (Figure 1). During the conducted study, an increase in the number of cases in March 2021 was observed, which may be identified as the third wave covering the period between 1 March and 30 April 2021 [6].

Responding to the increasing number of infections and deaths, the Polish National Health Fund issued a recommendation on limiting the number of planned procedures and personal consultations [11]. The adopted solutions, however, whose aim was to ensure the safety of patients and medical personnel, created a sense of limited access to medical care [12,13] and resulted in an increased interest among the society in non-medical methods supplementing health and immunity [14,15]. Dietary supplements started to be perceived as immunity-boosting products [16]—especially given the lack of effective methods of treatment [17]. Moreover, the good general condition that results from proper nutrition seems to be associated with a milder course of illness [18,19]. This is why, among other reasons, during the COVID-19 pandemic, Europeans willingly purchased simple or compound vitamin products, declaring their regular use in order to build immunity to pathogens [20]. Social media, as well as other mass media, played a key role, emphasizing the real role dietary supplements play in the prevention and treatment of coronavirus infections. The marketing circulation also produced misleading statements concerning the medicinal role of dietary supplements in cases of COVID-19 infections [21].

**Figure 2:**
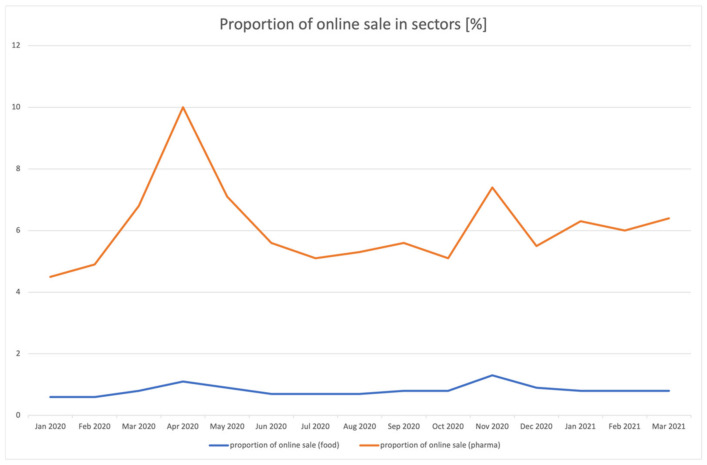
Share of online sale in the pharmaceutical and food sectors for January 2020–March 2021.

The subsequent waves of SARS-CoV-2 infections enlivened the e-commerce market in the pharmaceutical sector (Figure 2). The increased share of online stores in the sales of pharmaceuticals possibly also translate into an increase in the area of dietary supplements. Clearly smaller, but temporally coincidental increases were also recorded by the online food industry. In both cases, the increases may result from the subsequent phases of the so-called lockdown being introduced. In Poland, the first lockdown started on the 92nd day of the year, i.e., on 1 April 2020 [22], while the second stage of social and economic restrictions began in October–November 2020 [23,24].

**Figure 3:**
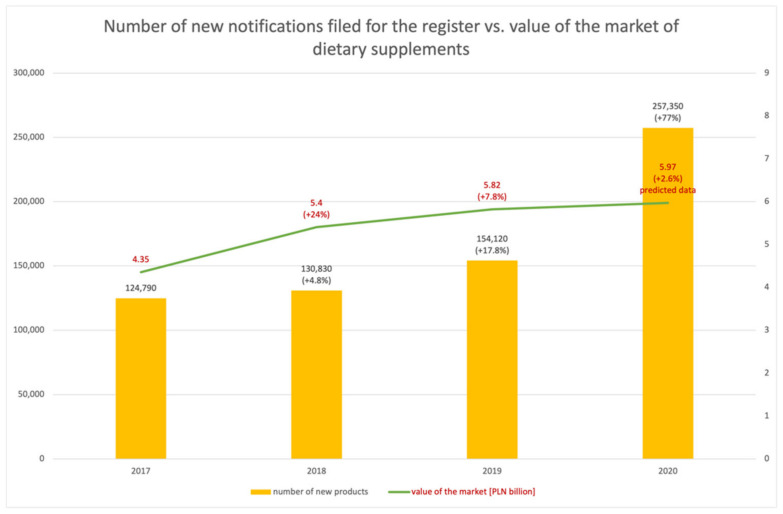
Number of new entries into the register of functional foods against the value of the market of dietary supplements in Poland.

The growth rate of new health-promoting products in the year of the pandemic turned out to be disproportionately higher than in the previous years—in 2020, it was as high as 77%. The predicted growth dynamics of the market of dietary supplements in Poland had been much lower, with the expected value for 2020 at 2.6% (Figure 3).

Considering the observations that unequivocally indicate the growing importance of dietary supplements in the diet of the Poles, it seems crucial to explore the nature of these changes, as well as to study the impact of the COVID-19 pandemic on the character of the new functional food products placed on the market, dietary supplements in particular.

The aim of this paper is to study the changes taking place in relation to the character and properties of new dietary supplements and functional food products notified as placed or planned to be placed on the market during the COVID-19 pandemic on the basis of data sourced from the register. To achieve our goal, we have made an innovative approach for data obtained from notional dietary supplements register. For the first time, detailed data on the compositions of dietary supplements notified in Poland have been analysed week by week, considering other variables related to the COVID-19 pandemic.

## 2. Materials and Methods

The starting material for the study was the register of dietary supplements and functional foods kept by the Chief Sanitary Inspector, described in art. 29 of the Act on food and nutrition safety. The analyses took into account the product filing date, the qualitative composition, and the data of the registering entity.

All products filed within the period were qualified for the study; these included preparations formally classified as dietary supplements, fortified foods, or special purpose foods, which will be hereinafter collectively referred to as dietary or food supplements, or —broadly—functional foods or health-promoting products.

On 4 February 2020, in relation to the Regulation of the Minister of Health amending the Decree on the format of a standard form for notification on products placed on the market for the first time on the territory of the Republic of Poland, the register of product types for which notification is mandatory, and the list of national research units competent to issue opinions entering into force, the manner and scope of information made available in the register changed—the day of filing of the notification of the first placement of the dietary supplement on the market made by the registering entity was made public, whereas in the previous years this information had not been publicly available; furthermore, it had not been possible to obtain it on application. The above change created the possibility of a new way of interpreting data from the register. Due to the information of the exact day of submitting notifications, we could analyse and compare registry data in the context of other data aggregated daily, weekly or in any other unit of time. It means especially seasons of the year, interest in Google searches and COVID-19 new cases reports. Such an approach was not possible before February 2020.

In the first phase of the analysis, 31,978 notifications from the period between 1 January 2020 and 19 March 2021 were taken into account, of which 96% were notifications concerning the placement or the intention to place dietary supplements on the market, while 4% concerned other foods for which registration is mandatory, including foods fortified with vitamins and minerals, among others. The above data were the basis for inferences on the regularities taking place in the period after the pandemic was announced. Then, using the Pareto principle, those ingredients that occurred in the studied period the most often were shortlisted. From among the 1257 ingredients occurring in the studied time interval a total of 426,808 times, only those that occurred in at least 1% of the studied products were included.

For comparative purposes, data from the same register for the year 2019 was used, taking into consideration the ingredients identified in the previous phase.

Data concerning the occurrences of the individual ingredients were expressed as the ratio between the occurrences of a given ingredient and the number of all the products registered in a given week of the year, i.e., the percentage of products in which the ingredient occurred was thus indicated.

The obtained information on the number of occurrences of the individual ingredients in newly registered products was compared with the data concerning the popularity of search terms pertaining to the ingredients shortlisted using the Pareto principle among Google browser users. For this purpose, the Google Trends tool was used—the “Interest in terms of time” report for Poland [25] for the period from 30 December 2018 to 21 March 2021 (rounded up to full weeks counted from Sunday).

The Google Trends report expresses interest (RSV—relative search value) in a given phrase on a scale from 0 to 100, the numbers denoting a lack of interest and peak popularity, respectively. As the Google Trends results are a relative measure, some aspect should be taken into consideration—the obtained values for specific ingredients searches may vary depending on the analysed period. To avoid some overinterpretation, we included Google Trends data for each ingredient since 2019. Such scope of data was helpful to distinguish some seasonal, repeatable trends from changes caused by the pandemic. For comparative purposes, in all the graphs, data from the report are placed on the Y axis and expressed as a percentage where 0% on the axis corresponds to 0 for the value from the Google Trends report—the same for the other values.

All the information concerning the frequency of use of the individual ingredients and their popularity in the Google browser was compared with the number of new confirmed COVID-19 cases. Data in this area were obtained from the publicly available reports published by the Polish Ministry of Health [26]. All the data were analysed in weekly intervals.

The shortlisted ingredients were also analysed in terms of marketing possibilities to attribute health-promoting properties to them by using health claims within the meaning of Regulation 1924/2006 [27] and on the basis of the list of such claims established by Regulation 432/2012 [28].

The day of 11 March 2020 was adopted to be the COVID-19 pandemic began (the 11th week of the year), i.e., the moment when the WHO Director-General announced at a press conference that with the growing number of cases and deaths, COVID-19 may be recognized as a pandemic [29]. The analysed data from the 11th week of 2020 were classified as sourced from the period after the announcement of the pandemic.

As far as the indicators of the frequency of use of the individual ingredients in dietary supplements are concerned, medians were calculated taking into account the periods before and after the announcement of the pandemic. Values from the period before the pandemic was announced and those from the period after its announcement were compared using the U Manna-Whitney test (with continuity correction) in Statistica 13 (Dell Inc.) software (Appendix A).

The analogic comparison was performed for the RSV parameter (Appendix B). Additionally, RSV results were analysed for potential correlations with the number of new SARS-CoV-19 infections using a Spearman’s rank correlation test (Appendix C).

The average number of ingredients in a single product in the years 2007–2020 was also determined, expressed as median. The calculations included 118,595 products entered into the register in the aforementioned period.

## 3. Results

### 3.1. Ingredients of Special Importance in the Register

Among the 1257 ingredients occurring in the products entered into the register in the period from 1 January 2020 to 19 March 2021, 66 occurred in less than 1% of the products.

**Figure 4:**
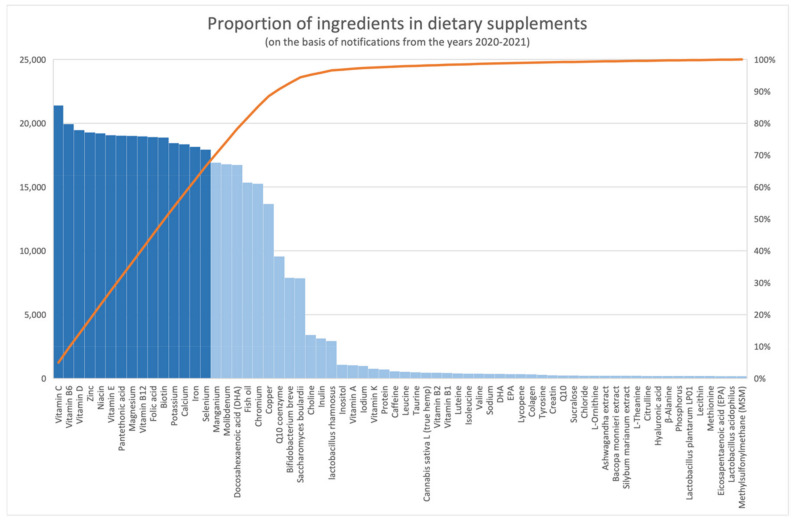
Proportion of ingredients in dietary supplements (based on entries from the period January 1 2020–March 19 2021).

Ingredients of special importance, which is understood as the frequency of their occurrence in the products and thus in the register, were vitamins C, B_6_, B_12_ D, E, as well as niacin, pantothenic acid, folic acid, biotin, and minerals such as zinc, magnesium, potassium, calcium, iron, and selenium (Figure 4), i.e., a total of 15 ingredients.

### 3.2. Average Number of Ingredients per Product

Based on the analysis of data available in the public register of functional food products placed on the market on the territory of the Republic of Poland, it can be stated that in the years 2007–2018 the average number of ingredients per product was slightly decreasing. In the years 2019–2020 the trend reversed, and a drastic increase was observed. Data for the year 2021 were disregarded due to the ongoing year.

**Figure 5:**
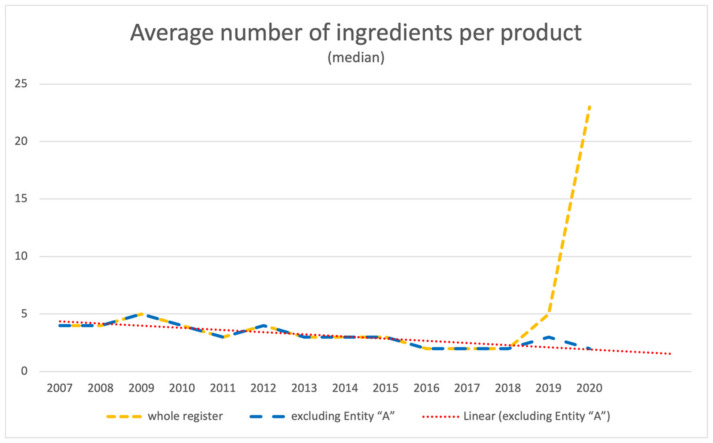
Average number of ingredients per product.

The increase in the number of ingredients per product observed since 2018 is connected with the activity of a single entity (hereinafter called Entity ”A”), which in the years 2018–2020 submitted 8%, 23%, and 57% of all the filed notifications, respectively. As shown in Figure 5, when data sourced from Entity ”A” are excluded from the analysis, the average number of ingredients per product expressed as median in the period in question is consistent with the predicted slightly decreasing trend line.

### 3.3. Activity of Registering Entities

**Figure 6:**
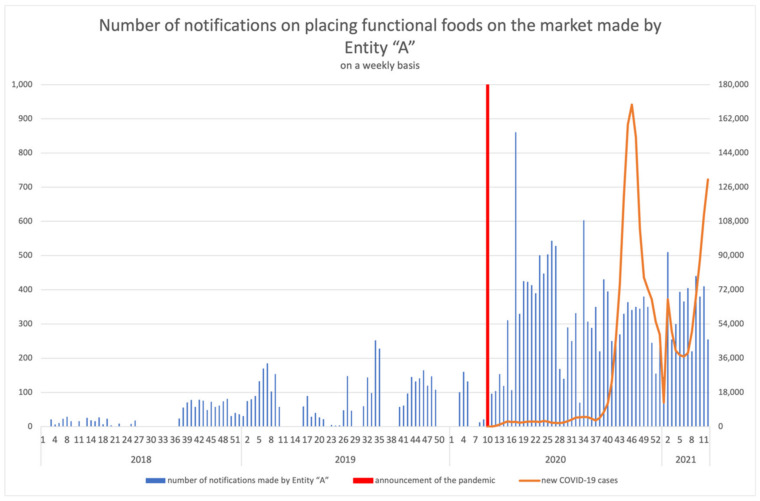
Number of notifications on placing functional foods on the market made by Entity “A” on a weekly basis, including the number of new COVID-19 cases in the years 2020–2021.

Considering the growth dynamics of the activity of Entity “A” in the register, an assessment of the weekly distribution of notifications on placing functional foods on the market made by the company was performed; in addition, for the years 2020–2021, the assessment included the number of new COVID-19 cases (Figure 6).

Apart from Entity “A”, which was the most active company in 2020 as far as the number of notifications made is concerned, the leading 10 entities in the category also included six that were active in the years 2017–2019 (Table 1). In 2020, four of those entities placed on the market, or notified on the intention to place on the market, the highest number of products compared to the previous years (Entities B, F, G, I). Two of the companies registered a smaller number of products than in the previous years; one company (Entity E) was characterized by a significantly lower activity in 2019 and 2021 compared to 2020; Entity D, however, registered a number of products in 2021 that constituted slightly over 2% when compared to the previous years. Entity J was only active in 2020.

### 3.4. Proportion of Ingredients in Products in the Period before and after the Announcement of the Pandemic and Their Popularity on the Internet

In all the analysed cases, the average proportion of the studied ingredients in products was significantly higher in the period after the announcement of the pandemic compared to the period before its announcement (Appendix A).

#### 3.4.1. Vitamin C and Zinc

**Figure 7:**
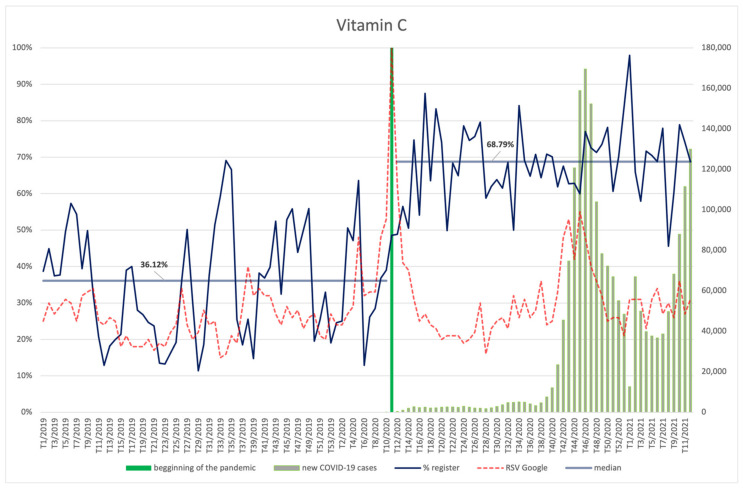
Comparison between the number of new registrations of products containing vitamin C, the number of COVID-19 cases, and the frequency of search for information on the ingredient in the Google browser.

**Figure 8:**
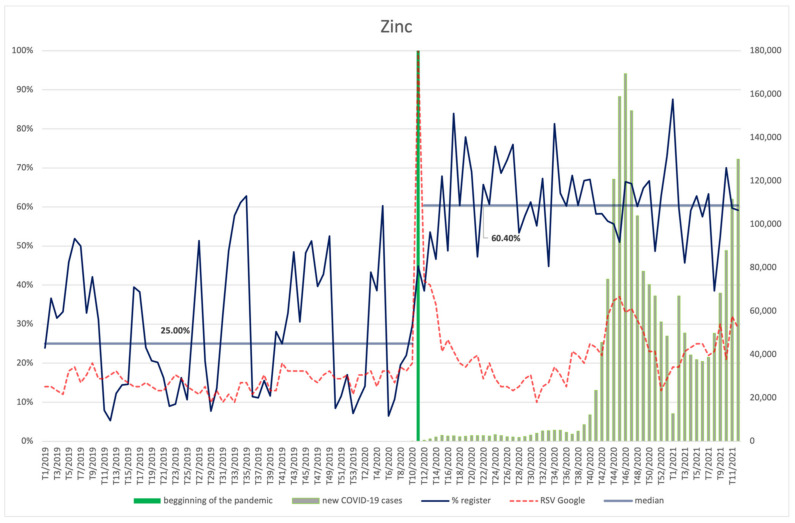
Comparison between the number of new registrations of products containing zinc, the number of COVID-19 cases, and the frequency of search for information on the ingredient in the Google browser.

Phrases pertaining to vitamin C (Figure 7) and zinc (Figure 8) were the most popular among the Internet users who used the Google browser in the week when the pandemic was announced (11th week of the year). Despite the initially high interest in the ingredient in question, its popularity 3 weeks after the announcement of the pandemic declined to the level from before the announcement. However, an increase in the relative search value (RSV) for search terms connected with vitamin C is again clearly visible starting from the 39th week of 2020, with a subsequent second highest peak, at 55, occurring in the 45th week, which corresponds to the second wave of infections in Poland.

Four to six weeks after the peak popularity of the search term, the accumulation of notifications on new products containing vitamin C as an ingredient takes place. The highest share of vitamin C in the products occurs in the 1st week of 2021, i.e., in the 8th week counting from the peak of new infections.

When compared to the period before the pandemic, a statistically significant (*p* < 0.001) increase of the average number of registered products containing vitamin C is observed—from 36.12% to 68.79% (Appendix B). There is also an average positive correlation between vitamin C RSV and the number of new COVID-19 cases (R = 0.29, *p* = 0.033).

As far as zinc is concerned, the distribution of new notifications on placing the products on the market is similar to that of vitamin C. The graph that reflects the interest in the ingredient is also similar after the March peak—the number of searches increases again in the 45th week of 2020 (second wave of infections), while in week 52, further increases of the indicator are recorded, coinciding with the beginning of the third wave. Despite these similarities, the increased interest of Internet users is statistically insignificant in the case of vitamin C, whereas it is statistically significant for zinc (*p* < 0.001). During the pandemic, interest in searching for information about zinc correlated positively with the number of COVID-19 new cases (R = 0.29, *p* = 0.031).

Moreover, the average share of products containing zinc is significantly higher (*p* < 0.001) after the announcement of the pandemic (60.40%) than before it began (25%). The periods of the highest share of products containing zinc coincide with the regularities observed for vitamin C.

#### 3.4.2. Vitamin D

**Figure 9:**
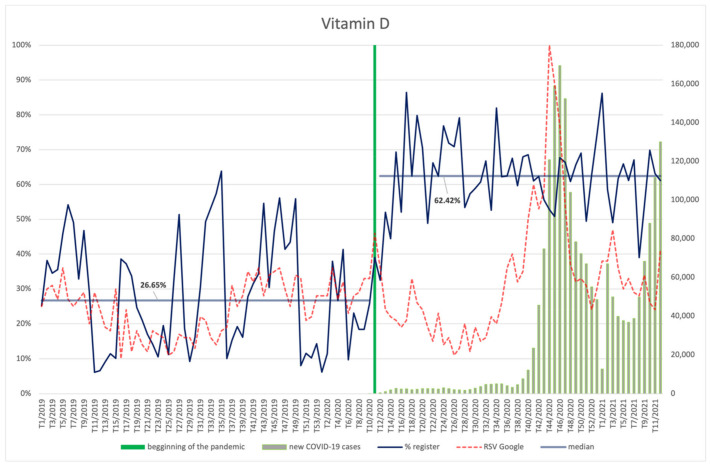
Comparison between the number of new registrations of products containing vitamin D, the number of COVID-19 cases, and the frequency of search for information on the ingredient in the Google browser.

An increased interest in vitamin D occurs in the winter months (Figure 9). This is visible throughout the whole analysed period. However, an increase in the online interest in vitamin D during the peak of the third wave of infections was almost three times higher compared to the previous year. A statistically significant increase of the RSV indicator occurs after the pandemic is announced (*p* = 0.016) (Appendix B). Mentioned above interest was dependent on new COVID-19 cases, showing a robust, positive correlation with this parameter (R = 0.58, *p* < 0.001).

The highest share of the ingredient in new products in the period after the pandemic is announced occurs in the 17th week of 2020 and in the 1st week of 2021. In the calendar year preceding the pandemic, periods of the highest share of products containing vitamin D occurred in weeks 6, 27, and 34. The average number of products containing the ingredient in question was significantly higher (*p* < 0.001) after the pandemic was announced (62.41%) than before (26.65%), twice reaching a value exceeding 86%—in the 17th week of 2020 and in the first week of 2021.

#### 3.4.3. Selenium

**Figure 10:**
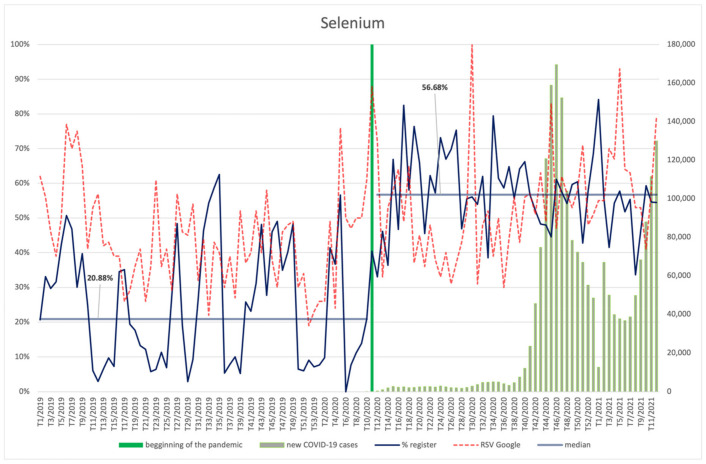
Comparison between the number of new registrations of products containing selenium, the number of COVID-19 cases, and the frequency of search for information on the ingredient in the Google browser.

The interest in selenium (RSV indictor) in the year preceding the COVID-19 pandemic was on a downward trend (Figure 10). At the beginning of 2020, the number of searches of the search term “selenium” started to increase, with a clear leap in the week when the global pandemic was announced. In the analysed period, Internet users’ interest in selenium was higher during the pandemic compared to the previous period, with a constant slightly increasing trend (*p* < 0.001) (Appendix B). It was also dependent on the number of new COVID-19 cases, showing an average, positive correlation with this variable (R = 0.31, *p* = 0.025). Peak RSVs for selenium are observed in the 30th week of 2020. No relationship between RSVs and the number of infections was observed.

#### 3.4.4. Potassium

**Figure 11:**
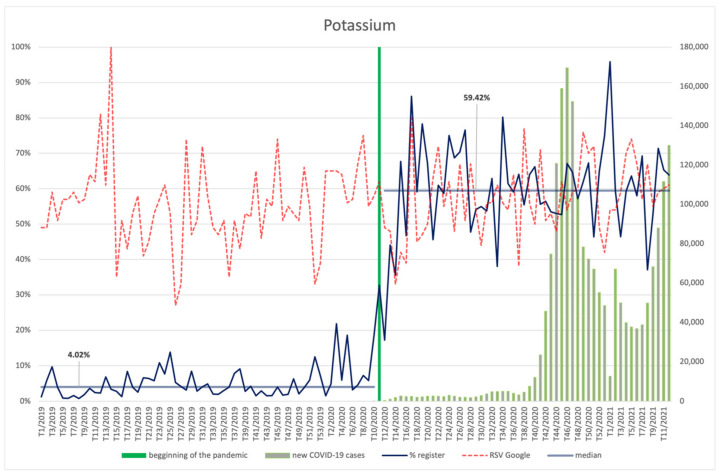
Comparison between the number of new registrations of products containing potassium, the number of COVID-19 cases, and the frequency of search for information on the ingredient in the Google browser.

The popularity of the search term “potassium” expressed as the RSV indicator remained at a similar level before and after the pandemic was announced (Figure 11). The highest RSV for potassium in the analysed period occurred in the 14th week of 2019. An average, positive correlation between searches and numbers of infections was observed (R = 0.29, *p* = 0.032). However, the increase in the number of filed products that contained potassium was statistically significant after the pandemic was announced (*p* < 0.001), i.e., from a low level of 4.02% up to 59.42%.

#### 3.4.5. Iron

**Figure 12:**
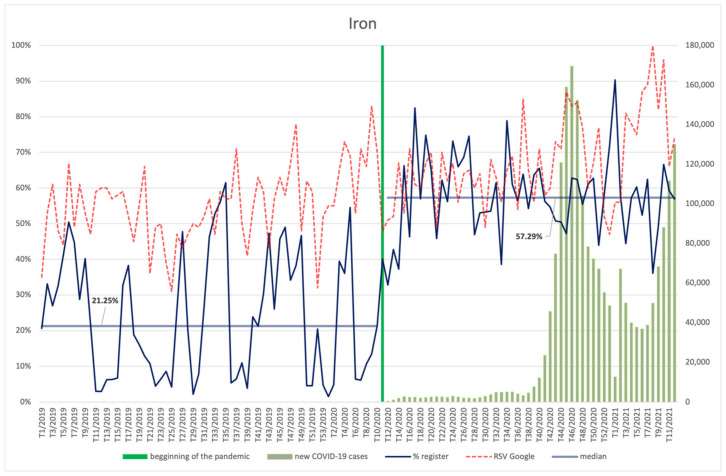
Comparison between the number of new registrations of products containing iron, the number of COVID-19 cases, and the frequency of search for information on the ingredient in the Google browser.

The interest in iron among Internet users slightly increases throughout the whole analysed period (Figure 12). The increase of the RSV indicator after the announcement of the pandemic is statistically significant (*p* < 0.001) (Appendix B). Peak RSV occurred in the 10th week of 2021. In general RSV corresponds (robust, positive correlation) to the increase in the number of infections (R = 0.50, *p* < 0.001).

An average of 57.29% of products entered into the register starting from the 11th week of 2020 contained iron, while for the previous period the indicator is significantly lower (*p* < 0.001) at 21.25%. Relatively, the highest number of newly registered products containing iron was recorded at the beginning of 2021 (the first week).

#### 3.4.6. Vitamin B6, Folic Acid

**Figure 13:**
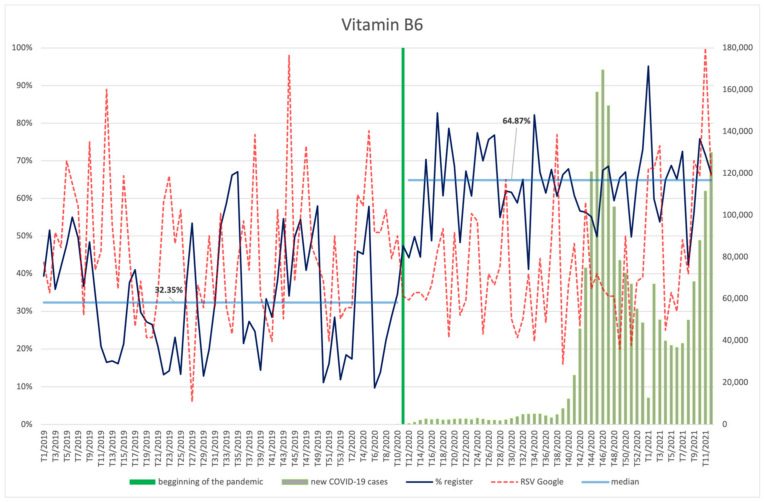
Comparison between the number of new registrations of products containing vitamin B6, the number of COVID-19 cases, and the frequency of search for information on the ingredient in the Google browser.

The frequency of searches for vitamin B6 (Figure 13) was on a downward trend starting from the beginning of the analysed period. Only towards the end of the period did an increase occur and in the 11th week of 2021 searches reached their peak value, which coincided with the beginning of the third wave of COVID-19 infections; however, there was no correlation in the analysed period.

After the announcement of the pandemic, vitamin B6 was used in new products significantly more often (*p* < 0.001) than in the period before the pandemic (64.87% vs. 32.35%).

**Figure 14:**
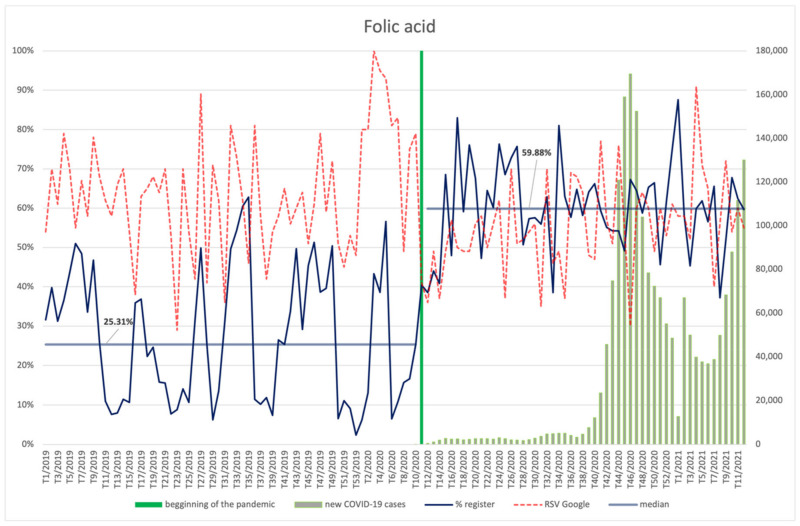
Comparison between the number of new registrations of products containing folic acid, the number of COVID-19 cases, and the frequency of search for information on the ingredient in the Google browser.

The interest in folic acid (Figure 14) among Internet users after the announcement of the pandemic decreased (*p* = 0.001) (Appendix B); however, during the pandemic, it correlated positively with the number of new COVID-19 infections (R = 0.35, *p* = 0.010). The highest interest in the ingredient was observed at approx. Nine weeks before the pandemic was announced. Otherwise, the occurrence of folic acid in newly registered products significantly increased after the pandemic was announced (*p* < 0.001), from over 25% to approx. 60%.

#### 3.4.7. Pantothenic Acid

**Figure 15:**
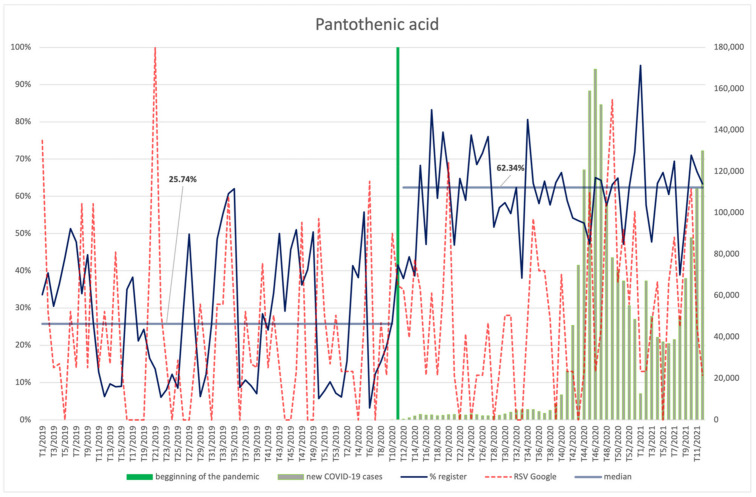
Comparison between the number of new registrations of products containing pantothenic acid, the number of COVID-19 cases, and the frequency of search for information on the ingredient in the Google browser.

The interest of Internet users in pantothenic acid (Figure 15) was at a similar level both before and during the pandemic, showing no significant correlation with the number of new COVID-19 infections. However, an interesting correlation between the increase in the number of searches, the shape of the second wave, and the renewed increase in the number of infections that constituted the beginning of the third wave can be observed.

Supplements containing pantothenic acid were registered significantly more often (*p* < 0.001) in the period after the outbreak of the pandemic (59.88%) than before (25.21%).

#### 3.4.8. Magnesium, Calcium

**Figure 16:**
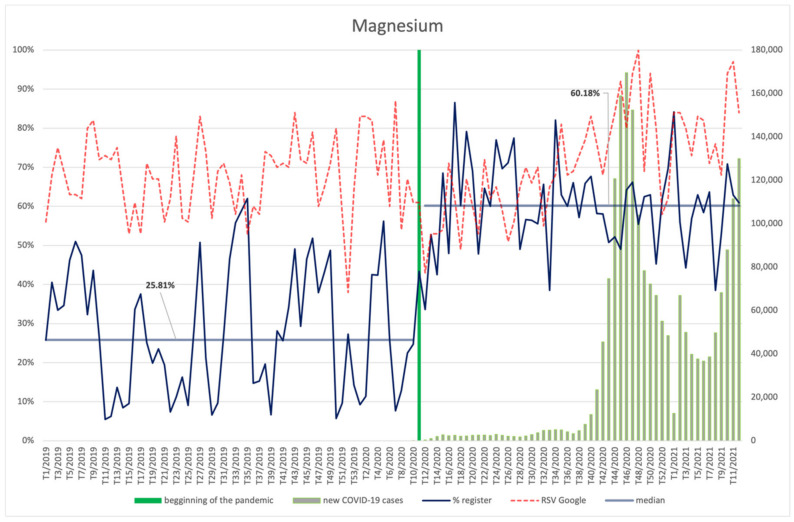
Comparison between the number of new registrations of products containing magnesium, the number of COVID-19 cases, and the frequency of search for information on the ingredient in the Google browser.

**Figure 17:**
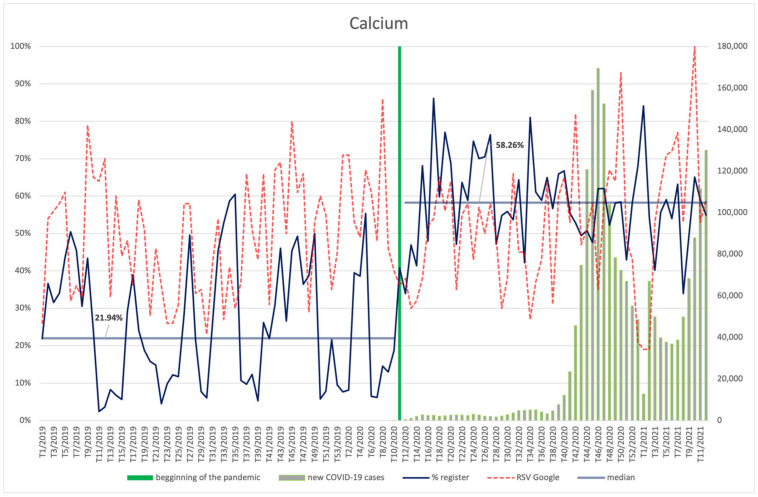
Comparison between the number of new registrations of products containing calcium, the number of COVID-19 cases, and the frequency of search for information on the ingredient in the Google browser.

Magnesium (Figure 16) and calcium (Figure 17) were characterized with a similar frequency of searches before and during the pandemic, with a slight increase in the latter period. Both magnesium and calcium RSV was dependent on the number of new infections, showing a robust, positive correlation in the case of magnesium (R = 0.75, *p* < 0.001) and average, positive correlation for calcium (R = 0.34, *p* = 0.012). The highest RSV was observed for magnesium in the 48th week of 2020, i.e., during the second wave of infections, whereas the interest in calcium peaked in the 10th week of 2021, i.e., it coincided with the increase in the number of infections that was the beginning of the third wave.

Both magnesium and calcium were included in newly registered products more often during the COVID-19 pandemic than before. The increase for magnesium was from 25.81% to 60.18% (*p* < 0.001); for calcium, from 21.94% to 58.26% (*p* < 0.001).

#### 3.4.9. Biotin, Vitamin E, Vitamin B12, Niacin

**Figure 18:**
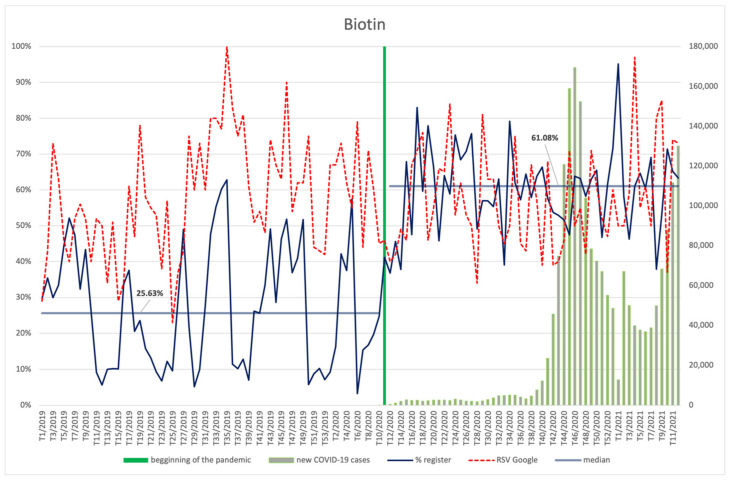
Comparison between the number of new registrations of products containing biotin, the number of COVID-19 cases, and the frequency of search for information on the ingredient in the Google browser.

**Figure 19:**
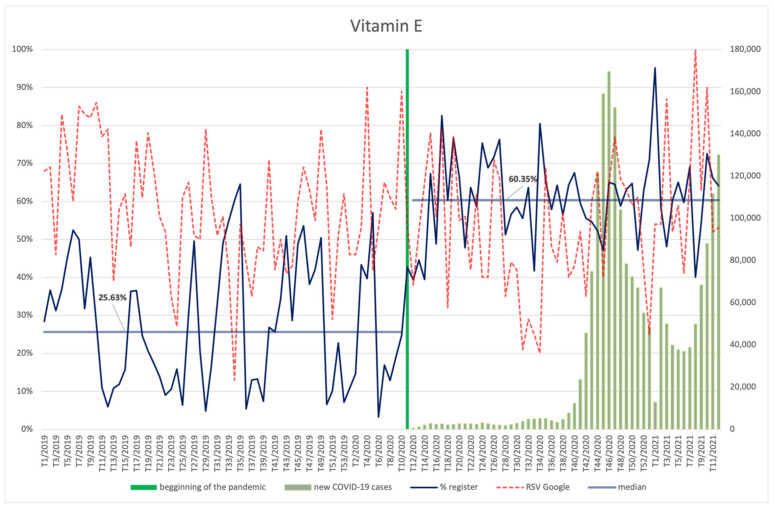
Comparison between the number of new registrations of products containing vitamin E, the number of COVID-19 cases, and the frequency of search for information on the ingredient in the Google browser.

**Figure 20:**
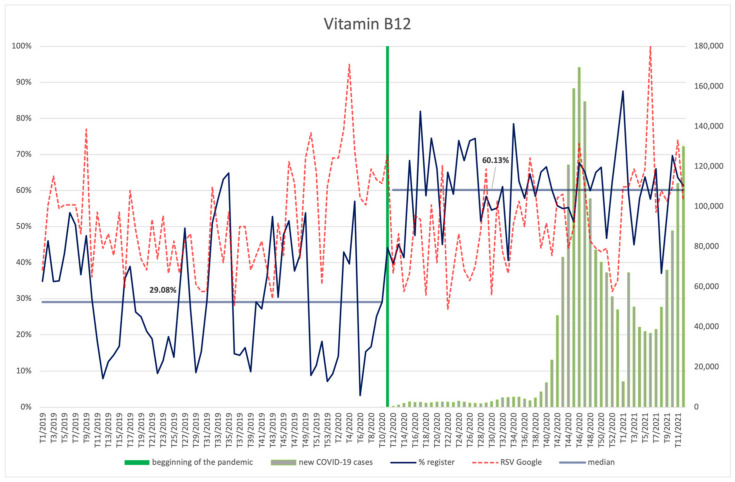
Comparison between the number of new registrations of products containing vitamin B12, the number of COVID-19 cases, and the frequency of search for information on the ingredient in the Google browser.

**Figure 21:**
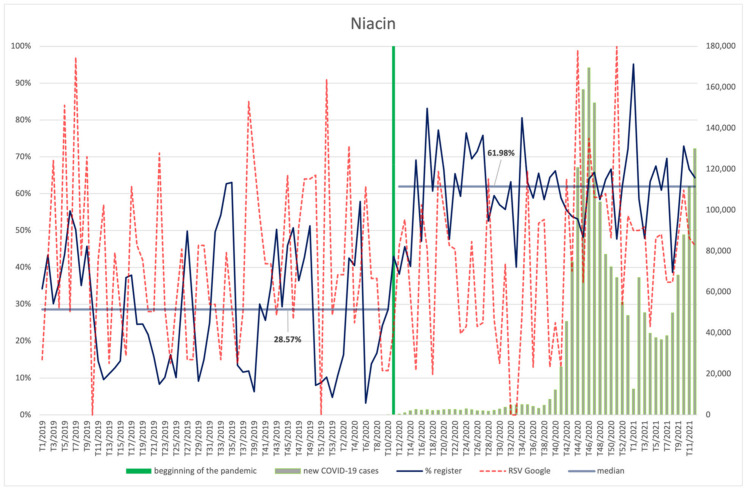
Comparison between the number of new registrations of products containing niacin, the number of COVID-19 cases, and the frequency of search for information on the ingredient in the Google browser.

The frequency of searches for biotin (Figure 18), vitamins E (Figure 19) and B12 (Figure 20), and niacin (Figure 21) performed by Internet users does not differ significantly between the period before the announcement of the pandemic and during its course. The values of the RSV indicator for niacin, including its maximum value, show correspondence with the number of new infections (R = 0.37, *p* = 0.006)—especially with the shape of the second wave, rising again as the third wave begins. Searches for vitamin B12 were also dependent on the new COVID-19 cases, showing an average positive correlation (R = 0.29, *p* = 0.035). No relationship with COVID-19 infections can be observed for the other ingredients. An interesting observation is the seasonal character of the interest in vitamin E (RSV indicator) in the analysed period of over two years. It reaches the highest values at around week 8 (February), then there is a drop, with the lowest values occurring around week 34 (August), followed by another increase in the next period.

Each of the products was filed for the register significantly more often (*p* < 0.001) during the pandemic than before: biotin (61.08% vs. 25.63%), vitamin E (60.35% vs. 25.63%), vitamin B12 (60.13% vs. 29.08%), and niacin (61.98% vs. 28.57%).

## 4. Discussion

The studies in the area of trends in dietary supplementation conducted thus far mainly covered consumer research, whose habits and beliefs concerning supplementation were tested scientifically. The results show that the consumption of supplements is a widespread phenomenon; their regular use was declared by over 38% of respondents [30]—with plant-based supplements frequently specified by those questioned [31]. Among students, vitamin-and-mineral preparations turned out to be particularly popular [32]. Such a widespread use of dietary supplements may lead to a supply of nutrients in quantities exceeding the tolerable upper intake levels (UL) [33], especially given that a tendency to supplement with several preparations at the same time can be observed among young, physically active people [34]. Consumer safety concerns connected with possible adverse effects of supplementation appeared parallel to the above conclusions [35]. These concerns may be exacerbated by the fact that marketing of supplements seem to be at odds with promotion of evidence-based knowledge [36].

In this study, the authors attempted to assess the market from the perspective of the activities of manufacturers and distributors of dietary supplements, as well as to study the impact of consumer behaviour online on the character of newly manufactured health-promoting products.

When compared to the results of studies from 2007 [31], despite the announcement of the pandemic, in the period from January 2020 to March 2021, supplements containing botanical ingredients (botanicals), used in the previous years to improve health and often to alleviate particular health conditions, were not among the most commonly used ingredients of dietary supplements and other health-promoting products. Among the products newly registered in the period, basic vitamins and minerals turned out to be the most popular. On the basis of the obtained results, it can be unequivocally concluded that among the new products notified to be placed on the market, those that dominated were compound products, containing a mixture of minerals and vitamins in various combinations and proportions. The character of the data, however, does not allow to conclude whether this state of affairs results from changes in consumer habits or rather is an attempt made by the manufacturers at shaping the market of health-promoting products based on relatively inexpensive and easily obtainable raw materials.

The legislative aspect also seems to be important as certain attractive marketing slogans can easily be used in relation to the analysed ingredients, those indicating a relationship between these ingredients and health.

What can be ascertained with a large dose of certainty, however, is that the compound character of products is compatible with consumer needs, who expressed their interest in preparations of this type also in the earlier years. It may be relevant in this context that a clear deterioration in the general quality of diet can be observed during the COVID-19 pandemic. This should be understood as extreme practices such as lowering the daily consumption of calories or, conversely, their excessive supply sourced from various types of foods—with increased consumption of alcohol in both cases [37,38]. In this respect, an adequate and deliberate supplementation of diet is crucial.

On the basis of the obtained results, the ingredients identified in the first part of the study may be divided into groups based on the popularity of their use by manufacturers. Typical for the beginning of the COVID-19 pandemic are certainly ingredients such as vitamin C, zinc, and selenium. Despite the fact that scientific studies do not confirm direct effectiveness of zinc or selenium in the treatment or alleviation of the course of COVID-19 [39], they are—similarly to vitamin C—substances that stimulate the immune system (Appendix D) [40]. Hence, consumer interest and the increased frequency of occurrence of these ingredients in newly introduced health-promoting products in the early stage of the pandemic seem to be justified.

Together with the so-called first wave of COVID-19 infections, the character of the main ingredients changed—potassium and biotin appeared in place of the typical vitamins and minerals stimulating the immune system. While the role of potassium—as discussed later—in the course of COVID-19 infections is based on medical evidence, the more frequent use of biotin by the manufacturers may result from the attempt to meet consumer needs in the area of skin and hair self-care. This may be an effect of the closing of the beauty sector during the period [41].

Pantothenic acid and—still—selenium may be considered as characteristic for this stage of the pandemic. The results of this study do not make it possible to unequivocally attribute vitamin E to any particular stage(s) of the pandemic. This ingredient showed a repeatedly seasonal character throughout the analysed period. An assessment of the influence of the COVID-19 pandemic on the interest in this ingredient and its popularity is therefore difficult. Its presence in products is connected with it being perceived as one of the many immunostimulants [42]. However, European law allows to describe vitamin E in health-promoting products only as a substance that protects against oxidative stress (Appendix D) [28].

A fundamental difference in the aforementioned relations was brought about by the second wave, during which ingredients such as vitamin D, magnesium, pantothenic acid, niacin, and—again—zinc, selenium, and vitamin C remained in the centre of interest of Internet users. To some extent, iron may also be included in this group, as its RSV indicator during the peak of the second wave of infections correlates with the number of cases; however, considering the fact that Internet users’ interest in iron increased throughout the whole studied period, it cannot be definitively proven that this seasonal increase is connected with the pandemic.

Observing the increase in new confirmed infections visible from approx. The 42nd week of 2021 may allow to conclude that the third wave was approaching, also characterized by its typical ingredients. These mainly include vitamins B6 and B12, magnesium, calcium, and iron, but also zinc. Vitamin B6 is the second ingredient, after vitamin C, most commonly used in functional foods placed on the market after the announcement of the pandemic, which may stem from the results that indicate its beneficial role in alleviating COVID-19 symptoms [43].

It turns out that the presented pattern of division of ingredients is not reflected in the data on the health-promoting products registered in the whole study period. As a rule, the discussed ingredients were used in newly registered products in similar proportions throughout the studied period—an increase in the frequency of use of one of them resulted in an increase in the use of the others. It would appear that differences in the proportions of particular vitamins and minerals in newly registered health-promoting products occurred mainly between the waves of the pandemic. This is particularly visible several weeks after the peak of the second wave, when peaks in supplement registration occur. This can be explained as a probable manufacturers’ answer to the rising numbers of infections.

Only the presence of potassium in products does not match the above pattern. Due to the fact that—as shown numerous times—potassium deficiency is connected with a considerably more severe course of a SARS-CoV-2 virus infection [44,45]; its presence in functional food products remained at an extremely high level since the pandemic was announced.

This leads to a general conclusion that companies creating new products during the pandemic tried to shape the market by delivering universal and multipurpose dietary supplements, rather than rely on seasonal consumer needs.

When comparing the above observations with the possibilities of attributing health-promoting properties to food ingredients in accordance with the legislative framework, it may be concluded that directly after the pandemic was announced Internet users searched for information on ingredients that have extreme immunostimulative properties. During the first wave, which was characterized by social restrictions (the so-called lockdown), rather than increasing numbers of infections, consumers searched for products that generally support the nervous system, including the cognitive and psychological functions, which coincides with the growing number of diagnosed cases of depression observed after the first wave [46]. Such consumer preferences persist until the end of the second wave.

Despite the described practices, the trend concerning the average number of ingredients in a single product was decreasing. It was reversed in 2018 by Entity “A”. Based on the description of the company posted on its official website, it can be determined that Entity “A” promotes and offers for sale the so-called personalized dietary supplements, i.e., those whose composition is matched individually through the process of a dietary interview that includes questions related to the health condition, directly before the purchase of the product. Such a form of activity explains the high increase in the number of new notifications registered in the official list of dietary supplements. This phenomenon is particularly visible in 2020, when Entity “A” was the leader in the number of newly registered products.

Only 411 out of the 1132 companies that filed notifications for the register in the years 2020–2021 were active in the earlier years, i.e., 2017–2019. Almost 64% of the entities that registered products for which notification is mandatory, including dietary supplements, did it for the first time or following a break in placing new positions from the discussed assortment group on the market that was longer than 3 years. Such a share of new entities in the chain emphasizes the importance of health-promoting products in the everyday diet of the Poles. At the same time, this may also indicate a demand for this group of products that is increasing during the pandemic.

An analysis of the collected data also showed that Entity “A”, which promotes the idea of personalized supplementation during its at least 2-year presence on the Polish market of dietary supplements, has not found its imitators.

## 5. Conclusions

The obtained results show that the COVID-19 pandemic contributed to changes in the market of dietary supplements directly after its announcement as well as during the individual phases of its course on the Polish market, which ranks among those with the highest value in Europe. Compared to 2019, the pandemic caused a considerable and significant increase in the proportion of the health-promoting ingredients identified in this article in the dietary supplements and other functional food products filed for the register in the studied period. The COVID-19 pandemic had a particularly significant impact on the increased share of potassium in dietary supplements, which is particularly interesting due to the lack of possibility of attributing marketing claims that would indicate supporting the immune system to the ingredient.

It is also clearly visible that the entity that registered the greatest number of new products in 2020 was creating—to a larger degree than before—the trend of multidirectional personalized supplementation. It was also observed that consumers searched for products with different properties during the different phases of the pandemic—first, those that supported the immune system and then those improving the nervous system.

## Figures and Tables

**Table 1:** Number of notifications on placing new functional food on the market made by the ten most active companies in the period January 2020–March 2021.

Year/Number of Notifications	Entity A	Entity B	Entity C	Entity D	Entity E	Entity F	Entity G	Entity H	Entity I	Entity J
2017			1285			51	127	362	74	
2018	1229	186	1722 (↑ 134%)			62 (↑ 122%)	62 (↓ 51%)	428 (↑ 118%)	77 (↑ 104%)	
2019	3558 (↑ 290%)	11 (↓ 94%)	730 (↓ 58%)		7	136 (↑ 219%)	63 (↑ 102%)	345 (↓ 19%)	68 (↓ 12%)	
2020	14448 (↑ 406%)	606 (↑ 5509%)	554 (↓ 24%)	325	314(↑ 4486%)	308 (↑ 226%)	154 (↑ 143%)	148 (↓ 57%)	131 (↑ 193%)	123
2021	4080	153	160	7	34	3	12	35	28	

## Data Availability

Publicly available datasets were analysed in this study. This data can be found here: https://powiadomienia.gis.gov.pl (accessed on 1 June 2021); https://arcgis.com/sharing/rest/content/items/b03b454aed9b4154ba50df4ba9e1143b/data? (accessed on 10 June 2021); https://www.arcgis.com/sharing/rest/content/items/153a138859bb4c418156642b5b74925b/data (accessed on 10 June 2021); https://trends.google.pl/trends/explore?date=2018-12-30%202021-03-21&geo=PL&q=witamina%20c (accessed on 19 April 2021); https://trends.google.pl/trends/explore?date=2018-12-30%202021-03-21&geo=PL&q=cynk (accessed on 19 April 2021); https://trends.google.pl/trends/explore?date=2018-12-30%202021-03-21&geo=PL&q=witamina%20d (accessed on 19 April 2021); https://trends.google.pl/trends/explore?date=2018-12-30%202021-03-21&geo=PL&q=selen (accessed on 19 April 2021); https://trends.google.pl/trends/explore?date=2018-12-30%202021-03-21&geo=PL&q=potas (accessed on 19 April 2021); https://trends.google.pl/trends/explore?date=2018-12-30%202021-03-21&geo=PL&q=żelazo (accessed on 19 April 2021); https://trends.google.pl/trends/explore?date=2018-12-30%202021-03-21&geo=PL&q=witamina%20b6 (accessed on 19 April 2021); https://trends.google.pl/trends/explore?date=2018-12-30%202021-03-21&geo=PL&q=kwas%20foliowy (accessed on 19 April 2021); https://trends.google.pl/trends/explore?date=2018-12-30%202021-03-21&geo=PL&q=kwas%20pantotenowy (accessed on 19 April 2021); https://trends.google.pl/trends/explore?date=2018-12-30%202021-03-21&geo=PL&q=magnez (accessed on 19 April 2021); https://trends.google.pl/trends/explore?date=2018-12-30%202021-03-21&geo=PL&q=wapń (accessed on 19 April 2021); https://trends.google.pl/trends/explore?date=2018-12-30%202021-03-21&geo=PL&q=biotyna (accessed on 19 April 2021); https://trends.google.pl/trends/explore?date=2018-12-30%202021-03-21&geo=PL&q=witamina%20e (accessed on 19 April 2021); https://trends.google.pl/trends/explore?date=2018-12-30%202021-03-21&geo=PL&q=witamina%20b12 (accessed on 19 April 2021); https://trends.google.pl/trends/explore?date=2018-12-30%202021-03-21&geo=PL&q=niacyna (accessed on 19 April 2021); https://stat.gov.pl/download/gfx/portalinformacyjny/pl/defaultaktualnosci/5466/14/75/1/tablice_sprzedaz_detaliczna_przez_internet.xlsx (accessed on 5 July 2021); https://stat.gov.pl/download/gfx/portalinformacyjny/pl/defaultaktualnosci/5466/14/72/1/tablice_sprzedaz_detaliczna_przez_internet.xlsx (accessed on 5 July 2021).

## Appendix A

**Table A1:** Comparison of the percent of products containing individual ingredients from the period before the pandemic was announced and from the period after its announcement using the U Manna-Whitney test (with continuity correction).

Ingredient	Before Pandemic			During Pandemic			*p* Value
	Median	Q1	Q3	Median	Q1	Q3	
Vitamin C	0.36	0.21	0.50	0.69	0.62	0.75	<0.001
Zinc	0.25	0.12	0.42	0.60	0.55	0.67	<0.001
Vitamin D	0.27	0.14	0.41	0.62	0.54	0.69	<0.001
Selenium	0.21	0.08	0.40	0.57	0.48	0.64	<0.001
Potassium	0.04	0.02	0.07	0.59	0.53	0.67	<0.001
Iron	0.21	0.06	0.38	0.57	0.50	0.64	<0.001
Vitamin B6	0.32	0.20	0.48	0.65	0.56	0.69	<0.001
Folic acid	0.25	0.11	0.40	0.60	0.54	0.66	<0.001
Pantothenic acid	0.26	0.10	0.40	0.62	0.53	0.66	<0.001
Magnesium	0.26	0.13	0.43	0.60	0.53	0.66	<0.001
Biotin	0.26	0.10	0.41	0.61	0.53	0.66	<0.001
Calcium	0.22	0.10	0.39	0.58	0.49	0.65	<0.001
Vitamin E	0.26	0.13	0.42	0.60	0.54	0.66	<0.001
Vitamin B12	0.29	0.15	0.43	0.60	0.55	0.66	<0.001
Niacin	0.29	0.13	0.43	0.62	0.53	0.67	<0.001

## Appendix B

**Table A2:** Comparison of the RSV of individual ingredients from the period before the pandemic was announced and from the period after its announcement using the U Manna-Whitney test (with continuity correction).

RSV	Before Pandemic			During Pandemic			*p* Value
	Median	Q1	Q3	Median	Q1	Q3	
Vitamin C	26.00	21.00	31.00	27.00	24.00	34.00	0.052
Zinc	15.00	14.00	18.00	22.50	17.00	28.00	<0.001
Vitamin D	25.00	18.00	30.00	29.50	21.00	37.00	0.016
Selenium	42.50	36.00	52.00	52.50	43.00	63.00	<0.001
Potassium	55.00	49.00	61.00	56.00	50.00	64.00	0.253
Iron	56.00	48.00	61.00	66.50	58.00	75.00	<0.001
Vitamin B6	45.50	31.00	57.00	37.00	30.00	50.00	0.088
Folic acid	62.50	54.00	72.00	55.00	49.00	61.00	0.001
Pantothenic acid	15.00	0.00	30.00	26.50	12.00	39.00	0.203
Magnesium	68.00	60.00	73.00	69.50	62.00	80.00	0.220
Biotin	56.50	45.00	73.00	55.00	46.00	67.00	0.818
Calcium	49.50	35.00	61.00	53.00	38.00	64.00	0.333
Vitamin E	59.00	48.00	69.00	54.00	41.00	65.00	0.117
Vitamin B12	50.00	41.00	61.00	50.50	40.00	60.00	0.978
Niacin	39.50	27.00	57.00	46.00	25.00	54.00	0.698

## Appendix C

**Table A3:** Correlation between Ingredient and the Number of COVID-19 New Infections.

Ingredient	Valid N	R Spearman	*p* Value
Vitamin C	54	0.29	0.033
Zinc	54	0.29	0.031
Vitamin D	54	0.58	<0.001
Selenium	54	0.31	0.025
Potassium	54	0.29	0.032
Iron	54	0.50	<0.001
Vitamin B6	54	0.07	0.604
Folic acid	54	0.35	0.010
Pantothenic acid	54	0.20	0.144
Magnesium	54	0.75	<0.001
Biotin	54	0.12	0.376
Calcium	54	0.34	0.012
Vitamin E	54	0.15	0.274
Vitamin B12	54	0.29	0.035
Niacin	54	0.37	0.006

## Appendix D

**Table A4:** List of health claims that can be attributed to the individual ingredients.

Ingredient/Health-Promoting Action	Immune System	Protection from Oxidative Stress	Beauty (Hair, Skin, Nails)	Reproductive Functions	Energy Metabolism	Nervous System	Psychological/Cognitive Functions	Preventing Tiredness and Fatigue	Bones and Teeth	Muscles	Cell Division	Blood and Circulatory System	Hormones	Mucous Membranes	Support of Ingestion of Vitamins and Other Nutrients	DNA Protection and Protein Synthesis	Acid-Base Balance	Vision Improvement
Vitamin C	•	•	•		•	•	•	•	•	•		•			•			
Zinc	•	•	•	•	•		•		•		•		•		•	•	•	•
Vitamin D	•								•	•	•				•			
Selenium	•	•	•	•									•					
Potassium						•				•		•						
Iron	•				•		•	•			•	•						
Vitamin B6	•				•	•	•	•				•	•					
Folic acid	•			•	•		•	•			•	•				•		
Pantothenic acid					•	•	•	•					•					
Magnesium					•	•	•	•	•	•	•					•	•	
Calcium					•	•			•	•	•	•						
Biotin			•		•	•	•							•		•		
Vitamin E		•																
Vitamin B12	•				•	•	•	•			•	•						
Niacin			•			•	•	•						•				
